# Emotional, inflammatory, and genetic factors of resilience and vulnerability to depression in patients with premenopausal breast cancer: A longitudinal study protocol

**DOI:** 10.1371/journal.pone.0279344

**Published:** 2023-02-14

**Authors:** Susana S. Almeida, Magda A. Oliveira, Rui Medeiros, Marina P. Guerra, Carmine M. Pariante, Lia Fernandes

**Affiliations:** 1 Psychiatry Service, Portuguese Oncology Institute of Porto (IPO Porto), Porto, Portugal; 2 Department of Clinical Neurosciences and Mental Health, Faculty of Medicine, University of Porto, Porto, Portugal; 3 Psychiatry and Psychology Service, CUF Porto Hospital, Porto, Portugal; 4 Psychology Service, Portuguese Oncology Institute of Porto (IPO Porto), Porto, Portugal; 5 Faculty of Psychology and Education Sciences, University of Porto, Porto, Portugal; 6 Molecular Oncology and Viral Pathology Group, Research Center of IPO Porto (CI-IPOP)/RISE@CI-IPOP (Health Research Network), Portuguese Oncology Institute of Porto (IPO Porto) /Porto Comprehensive Cancer Center (Porto.CCC), Research Center-LAB2, Porto, Portugal; 7 ICBAS, Abel Salazar Institute for the Biomedical Sciences, University of Porto, Porto, Portugal; 8 Biomedical Research Center (CEBIMED), Faculty of Health Sciences, Fernando Pessoa University (UFP), Porto, Portugal; 9 Research Department, LPCC- Portuguese League Against Cancer (NRNorte), Porto, Portugal; 10 Center for Psychology at University of Porto (CPUP), Porto, Portugal; 11 Department of Psychological Medicine, Institute of Psychiatry, Psychology and Neuroscience, King’s College of London, London, United Kingdom; 12 Psychiatry Service, Centro Hospitalar Universitário São João (CHUSJ), Porto, Portugal; Kasturba Medical College Mangalore, Manipal Academy of Higher Education, INDIA

## Abstract

**Background:**

Psychosocial stress and depressive disorder have been associated with cancer as putative contributors to worse prognosis. On the other hand, cancer diagnosis is a recognised life event that can contribute to distress and depressive states. Humoral and cellular inflammation can promote depressive disorder by means of decreased monoamine synthesis, glutamate neurotoxicity, neurogenesis and neuroplasticity, dysregulated hypothalamic-pituitary-adrenal axis, and glucocorticoid resistance. This protocol objectives are to observe the interactions between psychosocial variables and biochemical and immunological biomarkers in a longitudinal, prospective design; to identify inflammation-related depression endophenotypes in breast cancer patients and to understand if early diagnosed and treated depression in this population will translate in better inflammation status and better global prognosis.

**Methods:**

Prospective observational cohort, composed by 100 consecutive premenopausal patients, diagnosed with non-distant metastatic breast carcinoma and with no history of major psychopathology or other organic illness. The participants will have an in-person assessment in three different moments, along illness treatment and follow-up, with respect to cytometric, immunologic, and psychosocial parameters and will be tested for depression vulnerability and resilience inflammation-related functional genetic polymorphisms. Additionally, at years 5 and 10 post enrollment, patients`medical records will be assessed. As a control cohort, all patients excluded due to psychiatric history or past psychiatric treatments will have their clinical records assessed at years 5 and 10 after admission. All the data will be managed with the SPSS® software.

**Discussion and conclusion:**

This study is an original longitudinal cohort of breast cancer premenopausal patients, with a comprehensive approach to psychosocial, clinical, inflammatory, and genetic variables. It expects to provide evidence regarding the links between genetic, cytometric, immunologic, and psychosocial factors, their potential contribution to the pathophysiology of depressive disorder, breast cancer course, progression, and prognosis. It may further contribute with data to better efficacy of the psycho-oncological interventions.

**Trial registration:**

National Commission of Data Protection (CNPD) 13413/2017; Ethics Committee of IPOP project code CI-IPOP81/2017.

## 1. Background

Cancer diagnosis is experienced as a major chronic stressor whose response is modulated by genetic vulnerability, environmental variations, pharmacological issues, infectious responses, psychosocial stressors impact and adjustment processes [1, 2]. These psychosocial factors interact with the neuroendocrine and immune systems in a network that shares a common molecular language ensured by mediators and receptors called ergones (neurohormones, neurotransmitters and cytokines). Their role is to guarantee the mutual, functional, and multilateral involvement in complex interconnections and to enable a two-way signaling communication between both systems, having as ultimate goal the regulation of body reactivity and the maintenance of homeostasis [1].

There are growing evidence emphasizing the association between stress chronicity, cancer development and poorer prognosis through the maladaptive activation of the neuroendocrine pathways with resulting inflammatory response dysregulation [1–5]. A recent systematic review conducted by Cheng and Meiser [3] highlighted this interface between psychosocial indicators and neuroimmune systems, showing that higher scores of stress, and other negative psychosocial dimensions were correlated with biomarkers of worse prognosis and poorer health outcomes in cancer samples. However, on the contrary, data established an association between positive psychosocial variables and markers of favorable clinical and survival indicators in different tumor types. Previous studies noted that distinct negative and dysfunctional cognitive and emotional profiles were related with poor health outcomes and with the activation of distinctive physiological pathways translated into assorted implications in the disease processes [6–8]. Inversely, when positive, these psychological mechanisms play a crucial role in stress and negative emotions buffering, in resilience increasement, and in the exploration of more adjusted appraisals, meanings and coping strategies [2, 9, 10], that will also activate distinct physiological response patterns [6, 9, 11–15]. Cancer-related distress levels and resulting physiological outcomes do not depend on cancer condition *per se*, but from the patients`appraisals and assimilation/accommodation processes, that will impact on emotional and behavioral responses, and then rebound on emotion regulation and coping mechanisms [8–10, 16–20]. This set of intra and interpersonal psychological processes can also impact patients’ health behaviors, including sleep quality, nutritional habits, substance use, physical exercise, medication use, and social interactions frequency and quality. Literature has been clear in demonstrating that these are key components, since they are directly influenced by patients cognitive, affective, and behavioral experiences, and that, direct or indirectly, they can play a putative role on immune functioning [21–30].

The review performed by Cheng and Meiser [3] revealed that amongst the 14 psychosocial variables studied, depressive symptoms were the most frequently evaluated. Despite inconsistencies between the studies, the analysis showed a positive correlation with proinflammatory cytokines and a negative association with the anti-inflammatory cytokines.

### Inflammation and depression in cancer patients

Cancer diagnosis presents a distressful life event, impacting on physical and psychological suffering and unveiling fears about a future perceived as uncertain [31, 32]. As expected, cancer can be conceptualized as an important psychosocial stressor that can promote affective disorders, specifically depression [33, 34]. Major depressive disorder prevalence in cancer patients, excluding palliative care samples, is higher than the 4% described for the general population [35], with different studies suggesting it to be as high as 16,3%, using diagnostic interviews [36]. Importantly, studies have suggested that depressive disorder is associated with worse prognosis and higher mortality in cancer patients [33, 37–39].

The inflammatory theory of depression has reunited a body of evidence and it is believed that high sustained levels of pro-inflammatory cytokines, able to cross the blood-brain barrier (BBB), and glucocorticoid resistance, along with reduced neurogenesis can underlie depressive states and depressive disorder in patients with chronic medical illness, even in the absence of past history of depression [31, 40–44]. Clinical phenotypes associated with inflammatory markers have been described in epidemiological cohorts and an atypical depression symptom cluster, with a worse metabolic profile has emerged [45–47].

At cellular level, decreased plasmatic T_reg_ cells have been reported in depressive disorder, along with a reduction in anti-inflammatory cytokines TGF_ß_ and IL-10 [48]. Increased plasmatic T_reg_ cells may improve resilience to depressive disorder by their production of IL-4 stimulating astrocytes to produce BDNF, whilst promoting the conversion of meningeal monocytes and macrophages from a pro-inflammatory M1 phenotype to a less inflammatory M2 phenotype [49, 50].

Raised inflammation levels as measured by increased plasmatic C Reactive Protein (CRP) also have been shown and replicated in depressive patient samples, with recent data identifying a cut off value of CRP >3 mg L^−1^ associated with increased basal ganglia glutamate, correlating with anhedonia, and decreased psychomotor speed [51, 52]. Cancer patients who present with depression also have raised blood IL-6 and an increase in daily cortisol [53]. Authors have suggested that high IL-6 plasmatic levels are associated with faster tumor progression, reduced response to chemotherapy and decreased survival [37], along with a direct relationship between high stress status, depression, and abnormal social behavior [53, 54]. The treatment options in cancer can also affect the development and manifestations of depressive disorder, for example, when interfering with IL-2, IL-6, IFN-*α* or steroids which are well-known depression inducers [55–59].

### Functional polymorphisms of inflammation and depressive disorder

Major depressive disorder (MDD) has a multifactorial causation, with genetic factors contributing up to 40% [60]. Several genes associated with the inflammation, neuroplasticity, glutamate, and monoamine pathways have been suggested as contributing to the diversity of depression phenotypes and potentially modulating antidepressant response in humans [42, 61]. Genes associated with inflammatory cascades and particularly cortisol receptors have also been extensively studied, with some variants associated with MDD [62–65].

### Breast cancer, depression, and menopause

The incidence of depression in women is nearly twice as high as in men [66] and depressed females seem to experience younger onset and lower quality of life, with more physical symptoms than depressed males [67, 68]. Among many proposed theories, estrogens and progesterone appear to have a contribution. The incidence of depression appears higher and depression more severe in perimenopausal than in pre and postmenopausal women [69, 70]. Moreover, depression incidence after menopause becomes similar between genders and depressed premenopausal and perimenopausal women may present lower levels of estradiol [71, 72]. In agreement with this theory, premenopausal women have been described as better responders to serotoninergic antidepressants than men, and this difference fades out after menopause [73, 74]. Breast cancer is the most frequent cancer in women younger than 50 years and this group of patients present more frequent or more severe depressive symptoms and worse quality of life outcomes than their age-matched population without breast cancer and older breast cancer women [75]. In a prospective study, pre-menopausal breast cancer women going through chemotherapy, assessed for depression and quality of life by means of validated questionnaires, presented higher scores that postmenopausal patients [76]. Breast cancer treatment in younger patients may cause premature menopause, with sexual, fertility and sleep difficulties [75], all of which may have a contribution to the higher depression level and lower quality of life in this population [76, 77].

### Breast cancer, depression, and health outcomes

Patients with depressive disorders and breast cancer have been described with overall poorer health outcomes. Depression has been associated with cancer recurrence, all-cause mortality, and cancer-specific mortality [78]. It urges to better characterize the populations more likely to develop depression, aiming for a more prompt and effective treatment that could modify and improve their prognosis.

## 2. Materials and methods

### 2.1. Aims of the study

■ To measure the incidence of depression and its severity in breast cancer patients that endure premature menopause triggered by cancer treatment.
■ To observe the interactions between psychosocial variables and biochemical and immunological biomarkers since diagnosis and along patient’s treatment journey.
■ To understand the contribution of the past psychiatric history in the disease course by the comparison of the two cohorts at 5 and 10 years follow-up;
■ To identify inflammation-related depression endophenotypes in breast cancer patients, verifying if a particular profile confers increased risk (e.g. with lower counts of NK and CD8+ cells; shift from Th1 to Th2 cells; higher CRP, IL-6 and TNFα) or protection (e.g. higher IL-10, IL-4) against depressive symptoms development.
■ To establish if in our sample the genetic variants studied correlate with inflammation markers and adversity and if this effect offers protection or higher risk for depressive disorder development.
■ To understand if early diagnosed and appropriately treated depression in the journey of breast cancer patients through diagnosis and by-protocol treatments will translate in better inflammation status and better global prognosis.

### 2.2. Study design and population

This study was approved by the Ethics Committee of the Portuguese Institute of Oncology of Porto (project code CI-IPOP81/2017) and was registered on the National Commission of Data Protection (CNPD) code number 13413/2017.

This is a prospective naturalistic and longitudinal study protocol, with a sample composed by 100 consecutive premenopausal patients, screened according to the inclusion and exclusion criteria (Table 1).

**Table 1:** Study eligibility criteria at baseline.

Inclusion criteria	Exclusion criteria
Women recently diagnosed with stages I, II or III breast cancer	Any previous malignancy
Women who accept anticancer treatment according to the proposed protocol in the multidisciplinary team meeting	Prior major psychiatric history
Treatment protocol requiring chemotherapy	Previous cognitive symptoms or complaints
18 years or more	Other severe medical comorbidities (e.g., autoimmune diseases, chronic infectious diseases; severe metabolic syndrome)
Premenopausal age	Non available for follow-ups
Able to speak, read, and write Portuguese fluently	Refuse consent to retain questionnaires data
Willing and able to participate in the three assessment moments	Refuse consent to retain biological material (blood and saliva)
Written informed consent assigned after Ethical Committee approval

■ Attending the research complexity and the concern with rigorous methodologies this research is composed by two complementary sub-projects that focused together on genetic, cytometric, and immunological parameters and a broad of psychosocial indicators referred in literature as being potential contributors to the initiation, development, and cancer progression.

### 2.3. Patient recruitment method

#### 2.3.1. Main cohort

According to recent trends, for one year period the Breast Clinic at IPOP estimates to receive for treatment over 250 patients newly diagnosed with breast cancer, stages I-III, premenopausal (if eligible for hormonal therapy, this group will be normally prescribed tamoxifen, while postmenopausal patients are usually prescribed aromatase inhibitors). Therefore, over the estimated period of one year, the authors expect to evaluate approximately 100 consecutive women diagnosed with breast cancer, eligible according to the detailed inclusion and exclusion criteria. If they accept to participate, this experimental group will comprise all the patients meeting inclusion criteria. They will be signaled after the weekly breast cancer multidisciplinary professionals meeting and contacted by a research member who will present the study protocol and invite the patient to complete the assessment procedures.

To participate, the eligible individuals should accept the study conditions and fill in the written informed consent in the three evaluation moments.

Each participant must be evaluated for the first time after diagnosis but before the treatments beginning (excluding surgery). The recruitment started in 2017, finishing at completion of at least 100 patients’ full data gathering.

#### 2.3.2. Control cohort

The control group will be composed by a similar number of subjects to those included in the experimental group (approximately 100 patients), differing from the experimental group only due to the presence of past or present psychiatric history, or if under antidepressant treatment regardless not having a formal diagnosis of depressive or anxiety disorders recorded. They will be recruited concomitantly and consecutively with the experimental group and until the total number is achieved.

### 2.4. Data collection and biological sample banking

Each participant will be assessed in three different moments: baseline–after the diagnosis and before beginning chemotherapy (T1); at the end of chemotherapy (T2), approximately 3 to 6 months after T1, according to the chemotherapy schedule; and 6 to 8 months after treatments’ end (T3), depending on the nearest appointment with the physician.

In each of the three assessment moments, patients will be evaluated according to three different categories of parameters:

■ Clinical and sociodemographic parameters
■ Cytometric and immunological parameters
■ Psychosocial and behavioral parameters.

In the three evaluation moments, psychosocial and behavioral assessments will be collected at the same day of salivary cortisol and routine blood testing, always in the same sequence: salivary cortisol, blood sample, and psychosocial data.

To accommodate both ethical and literature-based recommendations, authors made options to conciliate the evaluation timepoints with the patients`predicted agenda of appointments and blood collection.

At T1 will be administered the *Mini-International Neuropsychiatry Interview* (MINI), a screening instrument to exclude patients with past and present mental illness, and collected the genetic testing sample, plus the procedures at T2 and T3.

Both main and control cohorts will have their medical records assessed at years 5 (T4) and 10 (T5) after enrolment or diagnosis. Data to be collected will be referrals to psychology or psychiatric consultations; psychiatric diagnosis; psychopharmacological and psychotherapeutic interventions; cancer recurrence and treatments history; blood tests and imaging reports.

#### 2.4.1. Data collection stages

**Fig 1:**
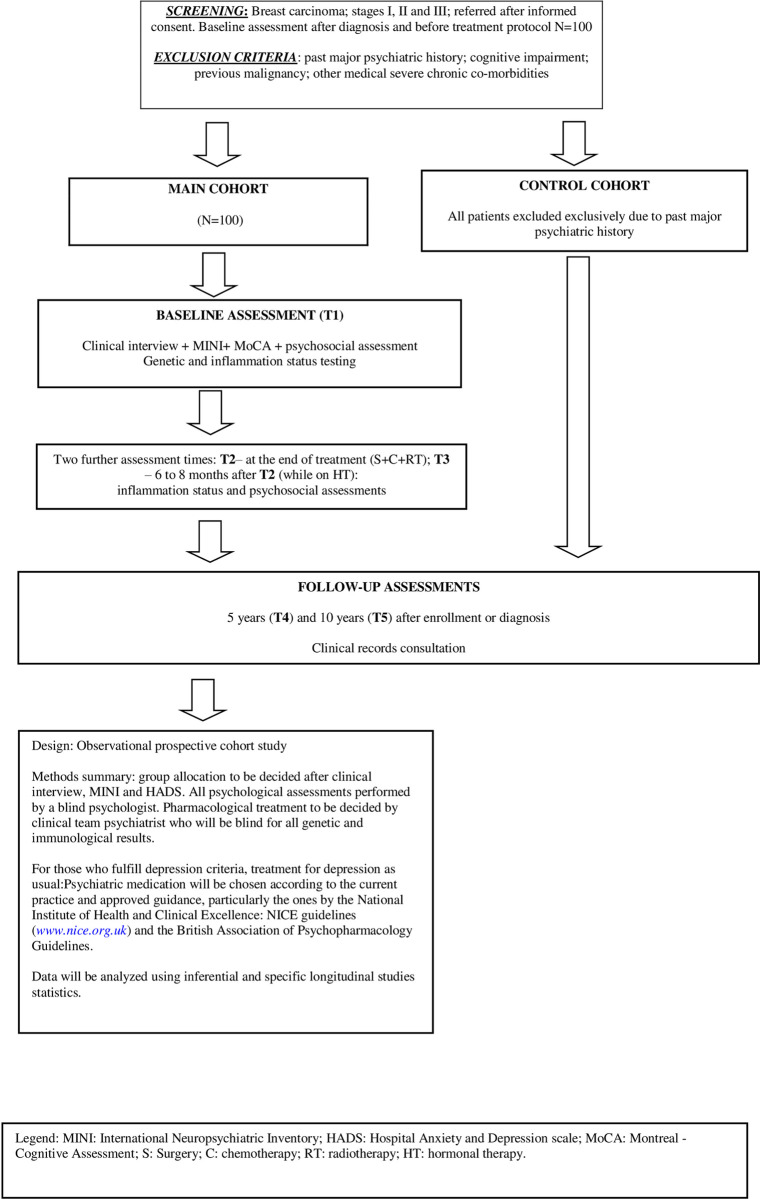
Study protocol flowchart.

The full trajectory and detailed steps involved in data collection and banking will be as follows:

■ After multidisciplinary team meeting the eligible patients will be contacted by phone for study presentation and to find out if they would be interested in participating. With those who agree, will be scheduled an appointment in the day in which they will attend to the hospital to perform the procedures before starting chemotherapy.
■ At the appointment, patients will receive complete information about the study protocol. Those who fully agree giving written informed consent will have the first blood sample for the protocol gathered (according to the medical procedures). The Salivette™ container for salivary cortisol testing will be handed over to the patients and collection procedure will be explained.
■ Over the following days (first assessment moment, T1), participants will come to the first chemotherapy session (maximum 15 days interval), will answer the psychobehavioral battery of questionnaires and will hand over the saliva sample (collected between 6am and 8am), to the lab for centrifugation and analysis.
■ Approximately 1 week before the second assessment moment (T2), patients will be contacted by phone to be reminded about the assessment date and that they will receive a second Salivette™ container by mail. A follow-up phone call will be made to confirm the receipt before T2 day.
■ At T2 (on average, two weeks after the end of chemotherapy treatment), participants will deliver the saliva collected in that morning, will give the second blood sample, and fill in the second battery of psychological evaluation questionnaires. The procedures with the blood and the saliva will be the same as in T1, except for genetic testing.
■ The procedures for T3 (6 to 8 months after T2) will be similar to T2.
■ Both main and control cohorts will have their medical records assessed at years 5 (T4) and 10 (T5) after enrolment or diagnosis (Fig 1).

#### 2.4.2. Depression and anxiety disorders treatment intervention procedure

Patients with complaints suggestive of mental health problems as identified by their oncologists or by HADS rating scale will be referred to psychological and/or psychiatric assessment as per current clinical protocol.

■ The patients clinically diagnosed with depression by the hospital psychiatrists, according to the ICD-10 criteria [79], will be treated with psychological cognitive-behavioral intervention and/or antidepressants depending on patient’s preferences and severity of the presentation.
■ Therapeutic options will be made according to the international state-of-the art and recommended *guidelines* and evidence for the treatment of depression [80].

### 2.5. Measures and materials

The biological and psychosocial measures, and their added value for current research are summarily introduced in Table 2.

**Table 2:** Summary of primary psychosocial and biological measures.

Method	Measure	Domain assessed	Reason for choice
Clinical interview	MINI Interview ^a^	Clinical diagnosis	Designed to yield reliable and valid neuropsychiatric diagnoses.
Face-to-face assessment	MoCA	Cognitive functioning screening	Short and reliable cognitive impairment screening tool. Differential diagnosis.
Self-report	HADS	Depression and anxiety symptoms severity	Specifical for physically ill patients Focuses only on psychological symptoms Suitable for patients in cancer;
	PSS	Perceived stress levels	Evaluate global and event-specific stress Assesses the perception of overloading, unpredictability, and uncontrollability
	Brief COPE	Coping mechanisms	Rapidly assesses multiple coping styles and skills Approaches the main coping mechanisms theoretically identified in literature
	TAS-20	Alexithymia	Widely used to measure alexithymia Suitable for clinical population
	PANAS	Positive and negative affect	Widely used measure Short and simple instrument that measures simultaneously positive and negative affect
	SRGS-SF	Personal growth	Evaluates different personal growth domains Nature of changes captured Short and. brief instrument Proper measure for patients with cancer;
	MLS	Meaning in life	Assesses a trait-like characteristic, being sensitive to disruptive events Short and brief instrument Suitable to evaluates physically ill patients`
	Life event checklist	Life stressors	Includes diverse potentially traumatic events Distinguishes the degree of exposure to the stressor
	FACIT ◾ FACT-G ◾ FACT-B ◾ FACT-Sp ◾ FACT-Cog	Well-being ◾ General well-being ◾ Breast cancer well-being ◾ Spiritual well-being ◾ Cognitive well-being	Specifically developed to measures health-related quality of life in cancer patients Includes different dimensions of general well-being Evaluates features related to wit cancer type (breast cancer) Approaches other cancer disease specific issues (spirituality; cognitive functioning/ impairment)
	PSQI	Sleep quality	Widely used instrument Short and quick measure while assessing different sleep domains
	ECS	(Un)healthy behavior	Evaluates and characterizes a set of distinct (un)healthy behaviors
Saliva collection	Diurnal salivary cortisol	Inflammation	Widely studied parameters
Peripheral blood	PCR CD4PE, CD3FITC, CD16+56PE, CD45PerCP, CD19PC7, CD8APC, CD14APC-H7, CD56FITC, CD25PE, CD127PC7	Humoral and cellular Inflammatory markers	Previous literature findings
Peripheral blood / Plasma	IL-2, IL-4, IL-6, TNF-α, IFNγ, IL-17a	Inflammation	Previous findings relating psychosocial factors and inflammation, namely in cancer patients
DNA extraction: GRS Genomic DNA Kit	Encoding genes, ^a^: rs1205, rs2794521, rs8193036, rs3819024, rs1800795, rs10499563, rs1800872, rs1800896, rs2243250, rs2227282 and rs1799964	Inflammation-related functional polymorphisms	Literature evidence showing to be related to the inflammatory pathway for depression.

*Note*. Measures will be assessed at all three timepoints unless marked with an ^a^ which indicate the measures that will only be collected at T1.

#### 2.5.1. A clinical and sociodemographic interview

A clinical and sociodemographic interview containing the following data will be completed:

■ Assessment moment and date
■ Definitive diagnosis and respective date, disease presentation, treatment protocol and its specific issues, treatments beginning and end dates, previous or current psychological and/or psychiatric monitoring

Patients’ characteristics (age, marital status, children, educational level, profession, professional status at the interview moment).

#### 2.5.2. Genetic parameters

Genetic variants were decided after a systematic review to elect the most consistent prevalent functional variants that can play a contribute to the inflammatory pathway for depression.

A peripheral venous blood sample (8 mL) will be collected from each subject enrolled in the study. After DNA extraction using the *GRS Genomic DNA Kit–BroadRange* (GRISP^®^), according to the manufacturer’s protocol, the polymorphisms in inflammatory markers encoding genes (rs1205, rs2794521, rs8193036, rs3819024, rs1800795, rs10499563, rs1800872, rs1800896, rs2243250, rs2227282 and rs1799964) will be analyzed by allelic discrimination using StepOnePlus™ qPCR Real-Time PCR machine (Applied Biosystems^®^).

The reaction is based on a 5’nuclease PCR assay, using a TaqMan assay, which includes two allele-specific TaqMan^®^MGB probes (Applied Biosystems^®^) containing distinct fluorescent dyes and a PCR primer pair to detect the specific SNPs. Real-time PCR will be carried out using a 6 μL reaction mixture, containing 1x Master Mix (Applied Biosystems^®^), with 1x probes (TaqMan^®^ assays: C_1747363_10; C_30370235_20; C_16176216_10: C_7479334_10; C_1799585_10; C_1839697_20; C_1747360_10; C_318207_10; C_7514871_10; C_16176374_10; C_11545877_10, Applied Biosystems^®^) and 20 ng of the DNA sample. Thermal conditions will be 95°C during 10 minutes for DNA polymerase activation, followed by 45 PCR cycles at 92°C for 15 seconds and 60°C for 1 minute. Quality control procedures implemented for genotype analyses will include double sampling in 10% of the samples to assess reliability and the use of negative controls to step-away false positives.

#### 2.5.3. Cytometric parameters

Quantification of total peripheral blood T-cells and lymphocytic sub-populations will be measured by means of *BD Trucount™ Absolute Counting Tubes* and with a monoclonal antibodies panel conjugated with flurochromes (CD4PE, CD3FITC, CD16+56PE, CD45PerCP, CD19PC7, CD8APC, CD14APC-H7, CD56FITC, CD25PE, CD127PC7). The samples will be acquired in cytometer *BD FACSCanto™* II and analyzed with the program *Infinicyt™*.

#### 2.5.4. Cytokines

Plasmatic cytokines IL-2, IL-4, IL-6, TNFα, IFNγ and IL-17a levels will be determined by protocol with the kit *BD™ Cytometer Bead Array Th1/Th2/Th17* (in pg/mL).

#### 2.5.5. Other biological measures

■ Peripheral blood high sensibility C Reactive Protein.
■ Diurnal Salivary cortisol collected with a Salivette™ container for salivary cortisol testing.

#### 2.5.6. Psychosocial and behavioral parameters

**The structured** *Mini-International Neuropsychiatry Interview* (MINI; [81]; Portuguese version [82]–This clinical assessment instrument will be administered only at baseline. This interview is designed to yield reliable and valid diagnoses of current and past history of psychiatric disorders according to DSM-5 criteria. In this study, it will be used to exclude patients presenting past or present psychiatric disorders.

A set of scales will be completed at each of the three assessment timepoints, always in the presence of a psychologist:

■ *Montreal Cognitive Assessment* (MoCA; [83]; Portuguese version [84])–This is a brief cognitive screening test for the identification of milder forms of cognitive impairment. It has an excellent sensitivity, utility, and accuracy for the identification of patients with Mild Cognitive Impairment (MCI) and Alzheimer’s Disease (AD) [84], for and cognitive impairment associated with other clinical conditions including tumors. Cognitive impairment associated with cancer or oncobrain is a well-known entity that may be accountable for symptoms also described for depression and anxiety disorders (e.g. concentration and attention difficulties). The MoCA will assist in the differential diagnosis between oncobrain and cognitive symptoms as part of depression or anxiety disorders that may develop [85].
■ *Hospital Anxiety and Depression Scale* (HADS; [86]; Portuguese version [87])–This is an effective self-report measure developed to screen affective disorders, specifically anxiety and depression, in the hospital settings or in somatically ill patients [86]. It is an accurate measure that excludes diagnostic criteria inherent to physical diseases and/or treatments performed and that emphasizes only psychological issues. Therefore, is considered suitable in the assessment of patients with cancer under treatment. It includes 14 items, equally distributed by anxiety and depression subscales. Each item is scored on a 4-point likert scale ranging from 0 to 3, resulting in a total that ranges from 0 to 21 in anxiety and depression scales [86, 87]. High scores indicate greater levels of anxious and/or depressive symptoms. The cutoff point for anxiety and depression for Portuguese population is 11 or above [87]. It also allows to stratify the anxiety and depression levels as mild, moderate, and severe. Alpha Cronbach`s for Portuguese adaptation are .76 and .81 for anxiety and depression respectively [87].
■ *Perceived Stress Scale* (PSS; [88]; Portuguese version by [89])–This is a standardized self-report questionnaire suitable to evaluate in what degree a person perceives her or his life as stressful, or how a person appraises her/his global stress in the aftermath of the exposure to a life stressful event. It is a good measure to evaluate both global and event-specific stress levels, with items designed to tap how unpredictable, uncontrollable, and overloaded a person finds one`s live [88, 89]. For being the most like the original version, and considering that it is not very extensive, it will be adopted the 14 items version. Items are rated on a 5-point likert scale, ranging from 0 (*never*) to 4 (*very frequently*), and total score can vary between 0 and 56. Higher scores indicate greater overall perceived stress. Cronbach`s alpha for original version was .86.
■ *Brief Cope Questionnaire* [90]; Portuguese version [91]–This inventory evaluates multiple coping styles and skills approached in the stress and coping, and self-regulation models [90, 91]. It is a short 28-item self-report questionnaire that rapidly measures 14 theoretically identified coping responses, each assessed with 2 items: Active Coping; Planning; Instrumental Support; Emotional Support; Religion; Positive Reframing; Self-Blaming; Acceptance; Venting; Denial; Self-distraction; Behavioral disengagement; Substances Use; and Humor [91]. Each item presents a coping thought or action that individuals may adopt under stress or in difficult situations. The questions are rated on a 4-point likert scale ranging between 1 (*I haven’t been doing this at all*) and 4 (*I’ve been doing this a lot*) with each subscale ranging between 2 and 8 [90, 91]. Higher scores indicate a higher prevalent in the use of the coping skill. The Portuguese validation presents an internal consistency ranging between .55 and .84 in the fourteen subscales [91].
■ *Toronto Alexithymia Scale* (TAS-20; [92]; Portuguese version [93])–This is a self-administered well-validated questionnaire widely used to measure alexithymia in clinical and non-clinical cohorts. It comprises 20 items rated on 5-point likert scale ranging from 1 (*strongly disagree*) to 5 (*strongly agree*) [92]. Total score ranges from 20 to 80 and higher scores express grater alexithymia characteristics. Portuguese cutoffs revealed that results equal or higher than 61 indicate alexithymia; scores between 60 and 52 correspond to a border area; and scores equal or inferior to 51 identify non-alexithymic subjects. The internal consistency of the Portuguese version is .80 [93].
■ *Positive and Negative Affect Schedule* (PANAS; [94]; Portuguese version [95])–This is a self-reported widely used measure developed to provide a short and simple instrument to characterize the presence of positive and negative affect in both clinical and nonclinical populations [94]. It includes two subscales with 10 items each, that list positive and negative affect respectively. Each item is rated on a 5-point likert scale, varying between 1 (*nothing or very slightly*) to 5 (*extremely*), with a total score fluctuating between 10 and 50 points in each of the subscales. Higher scores reflect a greater intensity of the affective response [94, 95]. The internal consistence of positive affect and negative affect subscales are .86 and .89 in the Portuguese validation [95].
■ *Stress-Related Growth Scale–Short Form* (SRGS–SF; [96]; Portuguese version [97])–This is a self-report scale that assesses the perception of growth and positive changes in different domains (personal resources, social relationships, life philosophy and coping skills) following a traumatic or threatening event [96, 97]. For being a brief measure, and for holding a balanced position regarding the nature of changes, not conceptualizing them illusional and short‐term benefits, nor deep and radical, this is considered a proper measure to assess patients with cancer [97]. Incorporates 15 items formulated after the instruction “*Because of this diagnosis/disease*, *I…*”. Each item is rated on a 3-point likert scale ranging between 0 (*not at all*) and 2 (*a great deal*), with a total score classified between 0 and 30 [96, 97]. Portuguese validation presented a reliability of .92.
■ *Meaning in Life Scale* (MLS; [98])–This is a single-dimension instrument that ascertains the perception of meaning in life as a trait-like variable, although assuming that this is not a static phenomenon, especially when facing an adverse life event such chronic and threatening physical conditions [98]. It comprises 7 items rated on a 5-point likert scale ranging between 1 (*I strongly agree*) and 5 (*I strongly disagree*), with a total score ranging between 7 and 35 [98]. The higher the score, the best the perception of meaning in life. Reliability coefficients in the validation study for the Portuguese population range between .74 and .78 [98].
■ *Life Events Checklist* [99]; Portuguese version [100]; checklist from the *Clinician-Administered PTSD Scale* (CAPS)–This is a checklist that includes 18 diverse potentially traumatic events. Respondents should answer according to if “*it happened to me*”, “I *saw happen*”, “*knew it happened*”, “*I’m not sure if it happened*” and “*does not apply*”.
■ *Functional Assessment of Chronic Illness Therapy* (FACIT; [101]). This is a modular questionnaire that measures specifically health-related quality of life in cancer patients. The Portuguese version includes a set of general questions that can be followed by a set of specific questions regarding cancer type or other cancer issues. Questions are rated in a 5-point likert scale ranging from 0 (*not at all*) to 4 (*very much*), and total score of each module is obtained adding all items or respective subscales scores. The higher the score, the greater the perceived well-being.

*Functional Assessment of Cancer Therapy–General Module* (FACT-G; [101]). Multidimensional tool that evaluates General Well-Being (GWB) through 27 items distributed over 4 subscales: Physical Well-Being (PWB; 7 items), Social Well-Being (SWB; 7 items), Emotional Well-Being (EWB; 6 items) and Functional Well-Being (FWB; 7 items). Should always be administered together with the specific modules. The original version presented an internal consistency of .90.

*Functional Assessment of Cancer Therapy–Breast Cancer Module* (FACT-B; [102]). One-dimensional measure that compiles 10 items extremely adequate to assess breast cancer specific concerns or particular aspects commonly experienced by these patients. Reliability ranges between .63 and .86 in the validation studies [102].

*Functional Assessment of Cancer Therapy–Spiritual Well-being Module* (FACIT-Sp; [103]). Questionnaire with 12 items equally distributed over sense of meaning, peace, and faith subscales.

*Functional Assessment of Cancer Therapy–Cognitive Module* (FACT-Cog; [104]). Evaluates cancer patients`perceived cognitive functioning through 37 items that focused on the perceived cognitive impairment (PCI), the perceived cognitive abilities (PCA), and the impact of perceived cognitive impairments on quality of life (IQL). Higher scores indicate less perceived impairment.

■ *Health Behaviors Scale* (ECS) (under validation)–This measure is composed by two instruments:

*The Pittsburg Sleep Quality Index* (PSQI; [105]); Portuguese version [106]–This is a self-administered questionnaire that measures sleep quality during the last month. It includes 9 questions that evaluate different components: subjective sleep quality, sleep latency, sleep duration, habitual sleep efficiency, sleep disturbances, use of sleep medication, and daytime dysfunction. Questions 5 to 9 are scored in a 4-point likert scale ranging between 0 and 3 [105, 106]. Higher scores reflect worst sleep quality. The reliability of the Portuguese version is .70.

*The Health Behaviors Scale* (ECS; Oliveira and Guerra, under validation)–Developed to assess different dimensions related with distinct health behaviors: diet and nutritional habits (17 questions), physical exercise (6 questions), substances use (coffee– 5 questions; tobacco– 7 questions; alcohol– 10 questions; drugs– 3 questions; medication– 3 questions), and leisure and social life (12 questions).

#### 2.5.7. Medical records

Data collected from medical records will gather referrals to psychology or psychiatric consultations; psychiatric diagnosis; psychopharmacological and psychotherapeutic interventions; cancer recurrence and treatments history; blood tests and imaging reports.

Protocol flowchart summarized in **Fig 1**.

## 3. Investigation`s subprojects

To cover all the objectives according to the latest literature and methodological rigor, this project was divided into two independent but complementary subprojects (A and B) that focus variables of different nature, having as common denominator the study of inflammatory modulation in breast cancer patients.

### 3.1. Project A

“Genetic resilience and vulnerability and its inflammatory modulation in depression in cancer patients” (Susana S. Almeida, Magda A. Oliveira, Rui Medeiros, Carmine M. Pariante, Lia Fernandes)

This project focuses particularly on the study of depression and anxiety in women with breast cancer, inflammatory modulation and its genetic factors of inflammatory resilience and vulnerability. Also aims to characterize possible oncobrain and fatigue phenomena as possible endophenotypes of inflammation-promoted and triggered depression and its implications on the quality of life and overall prognosis of patients with breast carcinoma. Lastly, with these data, it is a final goal to reflect about and identify the best treatment options for depressed patients with breast carcinoma.

### 3.2. Project B

"Psychoneuroimmunology and Cancer: The impact of psychosocial and behavioral variables on immune function and its consequences in the course of breast cancer (Magda A. Oliveira, Susana S. Almeida, & Marina Prista Guerra):

This project aims to study a set of psychosocial and behavioral variables pointed out in the literature as having a protective character, or in contrary, as having a crucial role in the genesis, development, and progression of cancer, through the compromise that they exert of the immune function. With these data more effective psychotherapeutic interventions can be planned to buffer suffering and potentiate positive dimensions of the disease–the final goal is to enhance physical and psychosocial outcomes.

All data presented in conferences and published peer-reviewed papers (for both subprojects) will integrate a reference to the sponsors and a warranty of the independence of the data obtained.

## 4. Planned statistical analyses

All the data will be managed with the SPSS^®^ v.27 and Process software and will be registered under the norms of the National Commission of Data Protection.

Firstly, data distribution will be analyzed according to the distribution by the normal curve, skewness (Sk) and kurtosis (K) in the three assessment timepoints.

The demographic and clinical data, as well as the biological and psychosocial measures characterization will be calculated by descriptive statistics.

The primary analysis will estimate the relationship between psychosocial variables (e.g. depression, stress, affectivity, meaning in life, well-being); between psychosocial indices and inflammatory biomarkers (e.g. anxiety and IL-6; stress and IFNγ; coping skills and pro or anti-inflammatory cytokines; positive affect and TNF-α; spiritual well-being and IL-2); and, between psychosocial factors and cytological parameters (e.g. stress/depression/unhealthy behaviors and the number of NK and cytotoxic T cells; positive affect/adjusted coping skills and cortisol).

Paired-sample *t* tests will be performed to compare the same subjects (psychological, immunological, and cytologically), between two assessment timepoints (T1 *vs*. T2; T1 *vs*. T3; T2 *vs*. T3).

Independent samples *t* tests will be estimated to compare two groups in relation to different variables (e.g. inflammation/stress/well-being in adjuvant and neoadjuvant cohorts; depression/negative affect/alexithymia/stress-related growth/well-being in patients with or without hormone treatment/immunotherapy; inflammation in patients practicing physical exercise or not; psychosocial / cytological / inflammatory indices according to the use of psychopharmacotherapy or not).

To compare three or more groups (nominal variables) in relation to the psychosocial, cytological or inflammatory variables, chi-squares will be estimated (e.g. differences in inflammation according to treatment protocols, marital or professional status).

Comparisons between three or more groups will be analyzed with the one-way ANOVA test (e.g. differences in the inflammatory or cytological markers according to the degrees of severity of depression, anxiety or alexithymic characteristics). Additionally, if necessary to control the effect or influence of an additional continuous variables (covariate–age, marital status, lifestyle, time since diagnosis) on the variance of a dependent variables, and to increase the power or sensitivity of the comparison between groups, ANCOVA will be performed.

One-way MANOVA tests will be calculated to compare groups in a range of different related characteristics that mirror a complex and dynamic reality (more than one dependent variable). This will allow to identify if there is a significant difference between two groups (e.g. adjuvant and neoadjuvant treatment; treatment with or without radiation) on the composite dependent variable when controlled the role of specific variables (e.g. anxiety, depression, mood, perceived stress, coping).

One-way repeated measures ANOVA will be performed the compare psychosocial (depression, affectivity, meaning in life, coping skills, well-being) and inflammatory (PCR, cortisol, cytokines) indicators over the three assessment timepoints.

Linear multiple regression models will be conducted to estimate how much of the variance of a variable (cytokines, cytotoxicity of NK cells, well-being) is explained by a model composed by a set (positive affect, meaning in life, personal growth, faith) of independent variables (number of factors calculated according to the sample`s size).

Hierarchical multiple regression will estimate how much an independent variable (e.g. positive affect, meaning in life, emotional support coping / depression, stress levels, alexithymia, substance use coping) adds to the prediction of a dependent variable (e.g. general well-being, pro-inflammation) after the previous variables have been controlled for (e.g. time since diagnosis or time elapsed since the end of treatment).

Process v3.5 by Andrew F. Hayes will be used to study possible moderation and mediation models between variables. Mediation models will be developed to test the hypothesis that the effect of a predictor variables under an outcome operates, fully or partially, through an intervening or mediator variable. Performed models will try to study the mediation that some psychosocial factors (e.g. positive affect, personal growth) exert under the relationship between a predictor (e.g. negative affect) and an outcome (stress, depression, breast cancer well-being); or, the mediation of some psychosocial factors (e.g. meaning in life, positive reframing coping, spirituality) under the relationship between a psychosocial variable (predictor–anxiety, depression, stress) and the inflammatory response (outcome–cortisol, PCR, interleukins). Covariate variables could be integrated in some of the models (e.g. age, type of treatment, sleep quality, nutritional habits, use of medication). Moderation models will be conducted to determine whether the relationship between two variables (dependent and independent variables) depends on, or is moderated by (in direction and/or strength), the behavior of a third variable (moderator). Models will be conducted to understand how a moderator influences or alters (e.g. psychopharmacological/psychotherapeutic interventions, marital status, leisure and social life) the direction and strength of the causal relationship between dependent and independent variables (e.g. depression–pro-inflammation; stress–cortisol levels; general well-being–anti-inflammatory levels).

Finally, after determining the genotypes for each of the polymorphisms for depression in DNA samples, the odds ratio (OR), 95% confidence intervals (CI 95%), and *p* values will be estimated using chi-square and Fisher Exact tests. The genotype frequencies will be compared with the frequencies of European and other international populations displayed at the Ensembl platform.

## 5. Anticipated results

### 5.1. Genomic outcomes

To assess in our sample a possible correlation of the most frequent functional genetic variants associated with inflammatory states with the likely expression of higher levels of inflammatory cytokines (and/or decreased anti-inflammatory ones) and of:

- Increased early adversity
- Worse coping mechanisms
- Depression and anxiety severity including impulsivity and suicidal behavior
- Worse cancer prognosis.

### 5.2. Cytological outcomes

Giving the literature data, authors expect to observe the following outcomes:

■ A decrease of the number of NK and cytotoxic T cells, including CD8+ cells and a shift in Th1 and Th2 cells, with higher expression of the latter, in patients with higher levels of stress, anxiety, depression, negative affect, alexithymia, dysfunctional coping styles and unhealthy health behaviors and a benefic role of positive affect, adjusted coping styles, meaning in life and stress related growth
■ Higher levels of cortisol associated with greater levels of stress, anxiety, depression, negative affect, alexithymia, dysfunctional coping styles and unhealthy health behaviors and, negatively related with the presence of positive affect adjusted coping styles, meaning in life and stress related growth.
■ An increased level of C-Reactive Protein positively associated with stress, anxiety, depression, negative affect, alexithymia, dysfunctional coping styles and unhealthy health behaviors scores, and negatively associated with positive affect, adjusted coping styles, meaning in life and stress related growth.

### 5.3. Immunological outcomes

According to what literature points out, authors expect the following outcomes:

■ A greater compromise of the immune function in patients exposed to higher levels of stressful life events
■ An immune suppression and potentiated inflammatory response in patients presenting higher level of stress, anxiety, depression, negative affect, alexithymia, dysfunctional coping styles and unhealthy behaviors
■ A protective immune response in patients with higher levels of positive affect, adjusted coping styles, meaning in life and stress related growth.

### 5.4. Quality of life outcomes

About quality of life authors anticipate the next outcomes:

■ Higher levels of emotional suffering (stress, anxiety, depression, negative affects), misadjusted coping styles, compromised emotional regulation patterns (alexithymia) and unhealthy behaviors are negatively related to general quality of life and specific breast cancer and spiritual quality of life.
■ The presence of positive affect, perceived personal growth and meaning in life is associated with greater levels of general and specific breast cancer and spiritual quality of life.
■ Higher fatigue scores and cognitive difficulties will be associated with higher inflammatory markers, and higher depression and anxiety scores, with lower quality of life.

### 5.5 Public health perspective outcomes

A more accurate approach, using genetic, inflammatory, and psychosocial data, to diagnosing depression in this specific group of patients, facilitating prompt and better-informed psycho-oncological interventions may jointly contribute to improving their physical and mental health global prognosis.

## 6. Discussion and conclusion

This study is an original longitudinal cohort of breast cancer premenopausal patients, with a set of comprehensive measures, including demographic, clinical, behavioural, psychosocial, inflammatory, and genetic variables, well recognized and internationally validated. This research will provide valuable information in one of the most promising fields for the Oncology and Psycho-Oncology: Psychoneuroimmunology. With the collected data will be possible to achieve a better understanding of the connections between psychosocial and behavioral stressors, the neuroendocrine factors, including treatment induced premature menopausal status, and immune functioning of breast cancer patients, from diagnosis throughout treatments. Data supply from functional genetic analysis will also contribute to enrich the knowledge about this complex myriad of interactions. The integration of all data and their longitudinal perspective will allow new insights for more effective and tailor-made psycho-oncological interventions (psychopharmacological and psychotherapeutic) with patients with breast cancer in particular, and in cancer patients in general. Findings may assist identifying increased risk endophenotypes for depression and anxiety disorders and selecting the psycho-oncological interventions with the highest preventive or remedial potential. Taken together, these may allow maximizing the patients’ psychological stability and consequently their adjustment to the disease and adherence to cancer treatment.

### Study limitations

This study presents the following limitations: firstly, the sample size will be relatively small due to budget and design choice constraints, to guarantee the well-controlled prospective nature of this study. Hence, the necessary control cohort only being studied at T4 and T5 by means of medical records data collection. Secondly, psychosocial, and behavioral data will be collected through self-report measures that can bring some bias. Thirdly, the observational nature of the design and the assessment procedures need to obey to ethical considerations that will introduce a gap of maximum of 2 weeks between the first blood and psychosocial data collection, and a time discrepancy amongst participants between the end of chemotherapy and the T2, and the T2 and T3 evaluations. Fourthly, cortisol collection will not be observer monitored, and therefore it is not possible to guarantee the schedule agreed for the collection.

### Study strengths

This is a naturalistic observational prospective long cohort, controlling a large psychosocial data set, including protective and risk factors for inflammation, anxiety, and depression. It is complemented by a control group in order not to exclude the possible contribution of major past psychiatric history in disease trajectory. It was designed to shed some light into the interplay between biological variables and their potential genetic determinants, population psychosocial characteristics and along the time frame from diagnosis to post treatment follow-up. These findings may add important information to the available data gathered from mostly cross-sectional studies on this subject, and open new perspectives for future research.
